# Liver receptor homolog-1 (NR5a2) regulates CD95/Fas ligand transcription and associated T-cell effector functions

**DOI:** 10.1038/cddis.2017.173

**Published:** 2017-04-13

**Authors:** Juliane Schwaderer, Ann-Kathrin Gaiser, Truong San Phan, MEugenia Delgado, Thomas Brunner

**Affiliations:** 1 Biochemical Pharmacology, Department of Biology, University of Konstanz, Konstanz, Germany

## Abstract

CD95/Fas ligand (FasL) is a cell death-promoting member of the tumor necrosis factor family with important functions in the regulation of T-cell homeostasis and cytotoxicity. In T cells, FasL expression is tightly regulated on a transcriptional level involving a complex set of different transcription factors. The orphan nuclear receptor liver receptor homolog-1 (LRH-1/NR5a2) is involved in the regulation of development, lipid metabolism and proliferation and is predominantly expressed in epithelial tissues. However, its expression in T lymphocytes has never been reported so far. Based on *in silico* analysis, we identified potential LRH-1 binding sites within the *FASLG* promoter. Here, we report that LRH-1 is expressed in primary and secondary lymphatic tissues, as well as in CD4^+^ and CD8^+^ T cells. LRH-1 directly binds to its binding sites in the *FASLG* promoter, and thereby drives *FASLG* promoter activity. Mutations in the LRH-1 binding sites reduce *FASLG* promoter activity. Pharmacological inhibition of LRH-1 decreases activation-induced FasL mRNA expression, as well as FasL-mediated activation-induced T-cell apoptosis and T-cell cytotoxicity. In a mouse model of Concanavalin A-induced and FasL-mediated hepatitis pharmacological inhibition of LRH-1 resulted in decreased hepatic FasL expression and a significant reduction of liver damage. In summary, these data show for the first time LRH-1 expression in T cells, its role in *FASLG* transcription and the potential of pharmacological inhibition of LRH-1 in the treatment of FasL-mediated immunopathologies.

Various immunological processes require a proper induction of programmed cell death by apoptosis, such as the elimination of neglected or autoreactive thymocytes, the clearance of virus-infected or altered target cells by cytotoxic lymphocytes or the regulation of effector T cells after an immune response. Deregulation of these apoptotic processes results in the development of chronic inflammation, autoimmune diseases, immunodeficiencies and tumor development.

Two major pathways are known to induce apoptosis: the intrinsic pathway controlled by Bcl-2 family members, and the extrinsic pathway initiated by death receptor activation.^1^ A prominent player in the death receptor pathway is Fas ligand (FasL/CD95L), which belongs to the family of tumor necrosis factor (TNF) family proteins. The biological activity of FasL is executed via binding to its cognate receptor Fas (CD95), which activates a caspase cascade and leads to apoptotic death in the target cell. FasL is expressed by various types of cells and tissues, but in particular by activated T cells and natural killer cells.^2^

After restimulation of previously activated T cells, FasL expression is rapidly induced, and the cell-autonomous interaction with the Fas receptor, or interaction with Fas on neighboring cells leads to apoptosis, which contributes to the homeostatic downregulation of T- and B-cell numbers at the end of an immune response.^3^ This process is referred to as activation-induced cell death (AICD) and peripheral deletion.^4^ Mutant mice with non-functional FasL as seen in *gld* (generalized lymphoproliferative disease) mice demonstrate increased numbers of autoreactive T and B cells, and associated pathologies, such as lymphadenopathies and autoimmune diseases.^5, 6^ Similar symptoms have been observed in ALPS (autoimmune lymphoproliferative syndrome) patients, which show genetic defects in the Fas signaling pathway, and sometimes also mutations in the *FASLG* gene.^7^

Another key effector function of FasL involves cell-mediated cytotoxicity. Primed CD8^+^ cytotoxic T cells, but also CD4^+^ T helper cells, rapidly express FasL or even release preformed and granule-stored FasL upon reactivation,^4, 8^ and interaction with the Fas receptor on target cells leads to their apoptosis. FasL-induced target cell killing appears to be involved in the induction of immunopathological disorders, such as T-cell-mediated hepatitis or Graft-versus-Host Disease.^9, 10, 11, 12^

FasL expression has to be tightly regulated in order to prevent uncontrolled tissue damage or inefficient immune cell depletion. In T cells, *FASLG* transcription is induced in naive and resting T cells upon T-cell receptor activation and involves the transcription factors NFAT (nuclear factor of activated T cells), NF*κ*B (nuclear factor 'kappa-light-chain-enhancer'), AP-1 (activator protein-1) and EGR-2 and -3 (early growth response protein).^2^ However, robust and rapid FasL expression is only induced upon reactivation of primed T cells. Primed proliferating T cells express c-Myc, which not only regulates cell proliferation, but also binds to specific binding sites in the *FASLG* promoter and thereby regulates *FASLG* transcription.^13, 14^

The orphan nuclear receptor liver receptor homolog-1 (LRH-1, NR5A2) is known to be highly expressed in tissues of endodermal origin, such as the intestine, liver, pancreas and ovaries.^15^ LRH-1 plays important roles in embryonic development, cholesterol and bile acid homeostasis and proliferation.^16^ LRH-1 has also been shown to indirectly regulate the immune system and associated inflammatory processes via the synthesis of immunoregulatory glucocorticoids in the intestinal crypts.^17^ Tissue-specific deletion or inhibition of LRH-1 and associated intestinal glucocorticoid synthesis consequently results in increased susceptibility to the development of intestinal inflammatory disorders.^18^

So far the expression and role of LRH-1 in the T-cell lineage has been unknown. Here we show that LRH-1 is expressed in CD4^+^ and CD8^+^ T cells, and is further induced upon T-cell activation. Furthermore, we identified LRH-1 binding sites in the *FASLG* promoter region, and demonstrate that LRH-1 is an important transcriptional regulator of FasL expression in T cells. Specific pharmacological inhibition of LRH-1 resulted in reduced activation- and LRH-1-induced FasL expression and cytotoxicity in T cells, and inhibited FasL-dependent liver damage in the context of experimental hepatitis *in vivo*. These data show that LRH-1 is a critical regulator of T-cell effector functions and that pharmacological inhibition of LRH-1 may represent a novel strategy in the treatment of FasL-mediated immunopathologies.

## Results

### LRH-1 expression in the T-cell lineage

**Figure 1:**
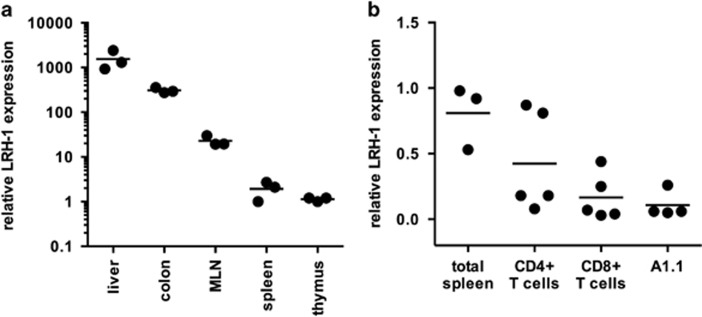
LRH-1 gene expression in lymphatic tissues and T cells. (**a**) Relative LRH-1 mRNA expression in liver, colon, mesenteric lymph nodes (MLN), spleen and thymus. Mean values (bars) from 3 wild-type C57BL/6 mice are shown. Each data point represents an individual mouse. (**b**) Relative LRH-1 mRNA expression in total spleen, sorted splenic CD4^+^ and CD8^+^ T cells and A1.1 T-cell hybridoma cells. Mean values (bars) and values of individual mice/cell samples (dots, *n*=3–5) are shown

Little is currently known whether LRH-1 is expressed in tissues of mesodermal or hematopoietic origin. We therefore analyzed LRH-1 expression in different organs of wild-type mice, and confirmed high LRH-1 expression levels in liver tissue and the colon. Interestingly, we observed low but detectable LRH-1 levels in primary and secondary lymphatic organs, such as the mesenteric lymph nodes, spleen and thymus (Figure 1a). LRH-1 was also detected in highly purified splenic CD4^+^ and CD8^+^ T cells (Figure 1b).

### *FASLG* is a direct transcriptional target of LRH-1

**Figure 2:**
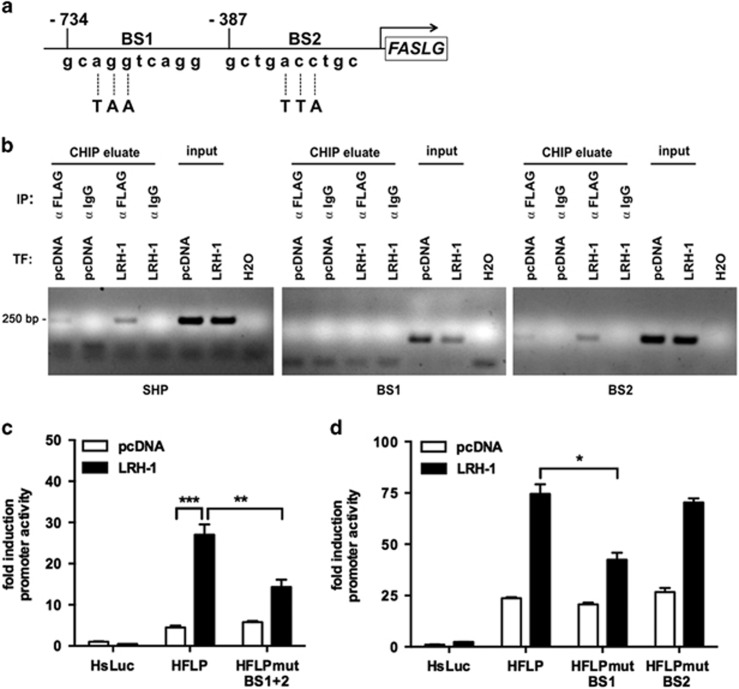
*FASLG* is a direct transcriptional target of LRH-1. (**a**) Schematic presentation of the two putative LRH-1 binding sites (BS1 and 2) in the *FASLG* promoter, and nucleotides mutated by site-directed mutagenesis. (**b**) Jurkat lT cells were transfected with control plasmid (pcDNA) or FLAG-tagged LRH-1 (LRH-1). Chromatin immunoprecipitation (ChIP) of LRH-1 was done using anti-FLAG antibodies or control IgG, and binding to the *SHP* promoter (SHP), or to BS1 and BS2 in the *FASLG* promoter was detected using sequence-specific PCR. IP, immunoprecipitation; TF, transfection. A typical experiment out of 3 is shown. (**c**) and (**d**) Jurkat lT cells were co-transfected with a control luciferase plasmid (HsLuc), the wild-type human *FASLG* luciferase promoter reporter (HFLP) or the reporter with mutated BS1, BS2 or both, and a control expression plasmid (pcDNA) or an LRH-1 expression plasmid (LRH-1). Luciferase reporter activity was measured and normalized to luciferase control plasmid. Mean values of triplicates±S.D. of a representative experiment (*n*=3) are shown (unpaired *t*-test; *P<0.05; **P<0.01; ***P<0.001)

To understand the potential role of LRH-1 in T cells, especially in the regulation of T-cell effector functions, we first screened for putative LRH-1 target genes using a bioinformatics approach. Our screening revealed two putative LRH-1 binding sites in the human *FASLG* promoter with the consensus sequence NN AGGTCA NN, one sense-orientated at position −734, and one anti-sense-orientated at position −387 (Figure 2a). We thus analyzed whether LRH-1 directly binds to these putative binding sites using chromatin immunoprecipitation (ChIP). *SHP* (Small Heterodimer Partner) is a known transcriptional target of LRH-1. In human Jurkat lT cells we found that LRH-1 was specifically precipitated from its binding site in the *SHP* promoter, in contrast to the negative IgG control (Figure 2b). Similarly, the sequence of the proposed binding site 2 (BS2) in the *FASLG* promoter could be specifically detected in the ChIP eluate, whereas detection of the binding site 1 (BS1) was inconsistent. These findings indicate that LRH-1 is binding specifically to the *FASLG* promoter.

Furthermore, we analyzed the role of LRH-1 in the transcriptional regulation of FasL expression using luciferase promoter reporter constructs. Co-transfection of Jurkat lT cells with an LRH-1 expression vector resulted in a significant activation of a 1.2 kb sequence of the human *FASLG* promoter (Figure 2c). In contrast, mutation of both LRH-1 consensus sequences in the *FASLG* promoter strongly reduced LRH-1-induced *FASLG* promoter activity. To study the importance of the individual binding sites single mutations in the *FASLG* reporter constructs were introduced (Figure 2a). Notably, mutation of BS1, but not BS2 resulted in significantly reduced *FASLG* promoter activity (Figure 2d). These findings indicate that LRH-1 directly regulates FasL expression via specific response elements in the *FASLG* promoter.

### Pharmacological inhibition of LRH-1 activity

As complete genomic LRH-1 deletion results in embryonic lethality,^19, 20, 21, 22^ a genetic model cannot be used to further address the role of LRH-1 in the regulation of FasL expression in T cells and associated functions. Recently, Benod *et al.*^23^ described a series of pharmacological inhibitors with LRH-1-specific inhibitory functions. Most notably, compound 3d2 showed strong LRH-1 inhibitory effect, whereas its close homolog steroidogenic factor-1 (SF1/NR5a1) was not affected. Thus, we further evaluated the application of this pharmacological inhibitor to study the role of LRH-1 in the regulation of FasL in T cells.

**Figure 3:**
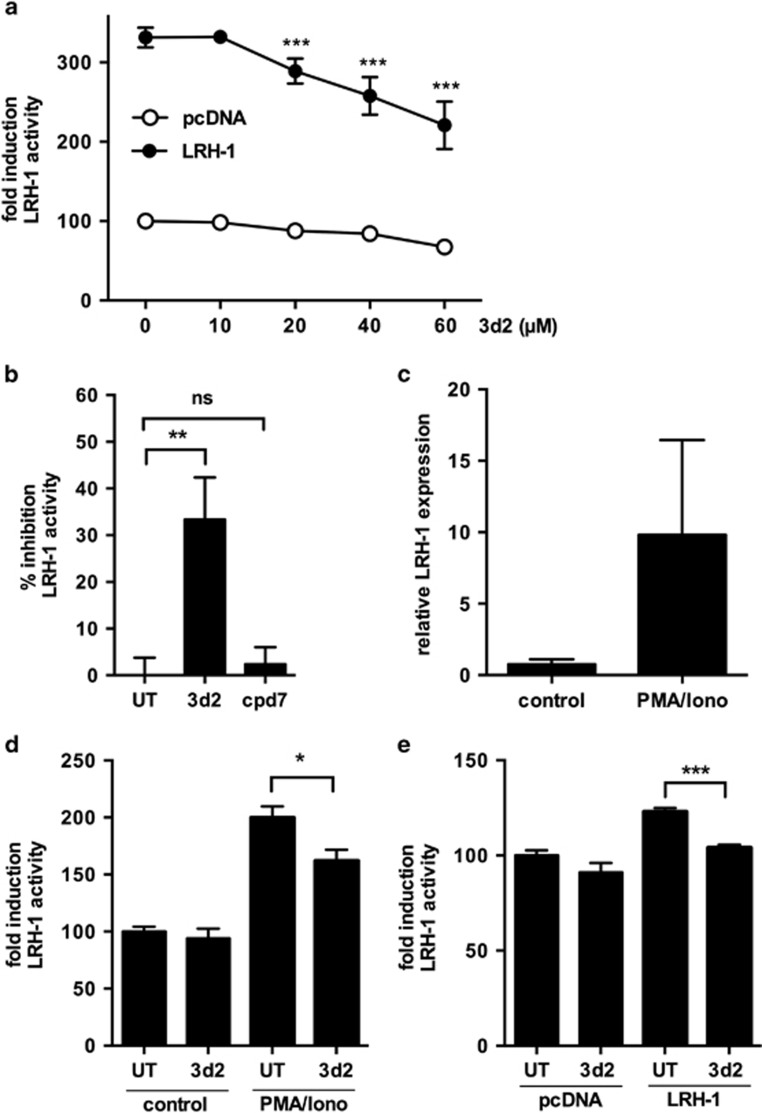
Pharmacological inhibition of LRH-1 activity. (**a**) HEK 293T cells were co-transfected with an LRH-1 luciferase reporter construct, and control plasmids (pcDNA) or LRH-1 expression plasmid. Cells were treated with increasing concentrations of 3d2. Luciferase reporter activity was measured and normalized to luciferase control plasmid. Mean values of triplicates±S.D. of a representative experiment (*n*=3) are shown (one-way ANOVA ***P<0.001). (**b**) HEK 293T cells were co-transfected with an LRH-1 luciferase reporter construct, and an LRH-1 expression plasmid or control plasmid (pcDNA). Cells were treated with buffer control (UT), 3d2 or cpd7 (60 *μ*M). Inhibition of the LRH-1 transcriptional activity by 3d2 and cpd7 is depicted as percentage compared with UT. Mean values of triplicates±S.D. of a representative experiment (*n*=3) are shown (unpaired *t*-test **P<0.01; ns, not significant). (**c**) Jurkat lT cells were stimulated with control buffer, or PMA (50 ng/ml) and ionomycin (500 ng/ml) for 18 h, and LRH-1 expression was analyzed by quantitative PCR. (**d**) Jurkat lT cells were transfected with an LRH-1 luciferase reporter construct, treated with control buffer (UT) or 3d2 (15 *μ*M), and stimulated with control medium or PMA/ionomycin (50/500 ng/ml) for 18 h. Luciferase reporter activity was measured and normalized to luciferase control plasmid. Mean values of triplicates±S.D. of a representative experiment (*n*=2) are shown (unpaired *t*-test *P<0.05). (**e**) Jurkat lT cells were co-transfected with an LRH-1 luciferase reporter construct, and control plasmids (pcDNA) or an LRH-1 expression plasmid. Cells were treated with 3d2 (15 *μ*M) or buffer control (UT) for 18 h. Luciferase reporter activity was measured and normalized to the luciferase control plasmid. Mean values of triplicates±S.D. of a typical experiment (*n*=2) are shown (unpaired *t*-test ***P<0.001)

In order to test the capacity of 3d2 in inhibiting LRH-1 activity, HEK 293T cells were co-transfected with an LRH-1 expression vector and an LRH-1 responsive reporter construct containing 5 repeats of an LRH-1 consensus sequence. As predicted, 3d2 showed a dose-dependent inhibition of reporter activity (Figure 3a). In contrast, the structural homolog of 3d2, compound 7 (cpd7), did not show any inhibitory effects on LRH-1 activity, confirming the specificity of 3d2 for LRH-1 (Figure 3b).

We next investigated the effect of pharmacological inhibition of LRH-1 activity in Jurkat lT cells. Phorbol myristate acetate (PMA)-induced MAP kinase activation has been reported to promote LRH-1 phosphorylation and activation of its transcriptional activity (Figure 3c).^24^ In agreement with this report we found that PMA plus ionomycin or LRH-1 overexpression-induced LRH-1 reporter activity, which was inhibited by treatment of cells with 3d2 (Figures 3d and e).

### Pharmacological inhibition of LRH-1 activity restricts *FASLG* promoter activity and expression

**Figure 4:**
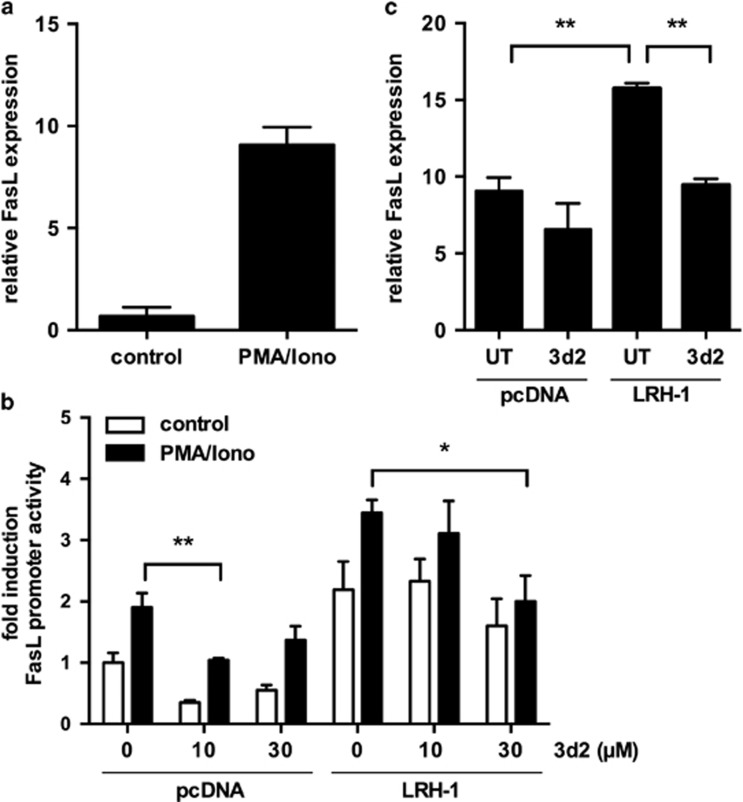
LRH-1 inhibition by 3d2 restricts FASLG promoter activity and expression. (**a**) Human FasL mRNA expression of Jurkat lT cells after treatment with PMA/ionomycin (50/500 ng/ml) for 18 h was determined using quantitative PCR. Mean values of triplicates±S.D. are shown. (**b**) Jurkat lT cells were co-transfected with a *FASL* luciferase promoter reporter, and control plasmids (pcDNA) or an LRH-1 expression plasmid. Cells were then stimulated with PMA/ionomycin (50/500 ng/ml) and the indicated concentrations of 3d2 for 18 h. Luciferase reporter activity was measured and normalized to luciferase control plasmid. Mean values of triplicates±S.D. of a representative experiment (*n*=3) are shown (unpaired *t*-test; *P<0.05; **P<0.01). (**c**) Jurkat lT cells were transfected with control plasmid (pcDNA) or LRH-1 expression plasmid. Cells were pre-treated with buffer control (UT) or 3d2 (20 *μ*M), and stimulated with PMA/ionomycin (50/500 ng/ml). FasL mRNA was measured by quantitative PCR. Mean values of triplicates±S.D. of a representative experiment (*n*=2) are shown (unpaired *t*-test; **P<0.01)

We next set out to test the effect of LRH-1 inhibition on activation- and LRH-1-induced FasL expression. Gene expression analysis demonstrates that FasL mRNA expression was strongly induced after activation by PMA plus ionomycin, mimicking T-cell receptor stimulation (Figure 4a). Overexpression of LRH-1 as well as PMA and ionomycin induced *FASLG* promoter activity (Figure 4b). The results showed that 3d2 treatment was able to inhibit the basal, activation-induced as well as LRH-1 overexpression-induced *FASLG* promoter activity in a dose-dependent manner. In addition, 3d2 treatment also decreased LRH-1-induced FasL mRNA expression, thereby confirming the 3d2-mediated inhibition of LRH-1 (Figure 4c). In summary, these findings demonstrate the pharmacological inhibition of LRH-1 by 3d2, which results in reduced activation- and LRH-1-induced FasL expression in Jurkat lT cells.

### LRH-1 inhibition blocks activation-induced FasL expression and associated T-cell suicide

**Figure 5:**
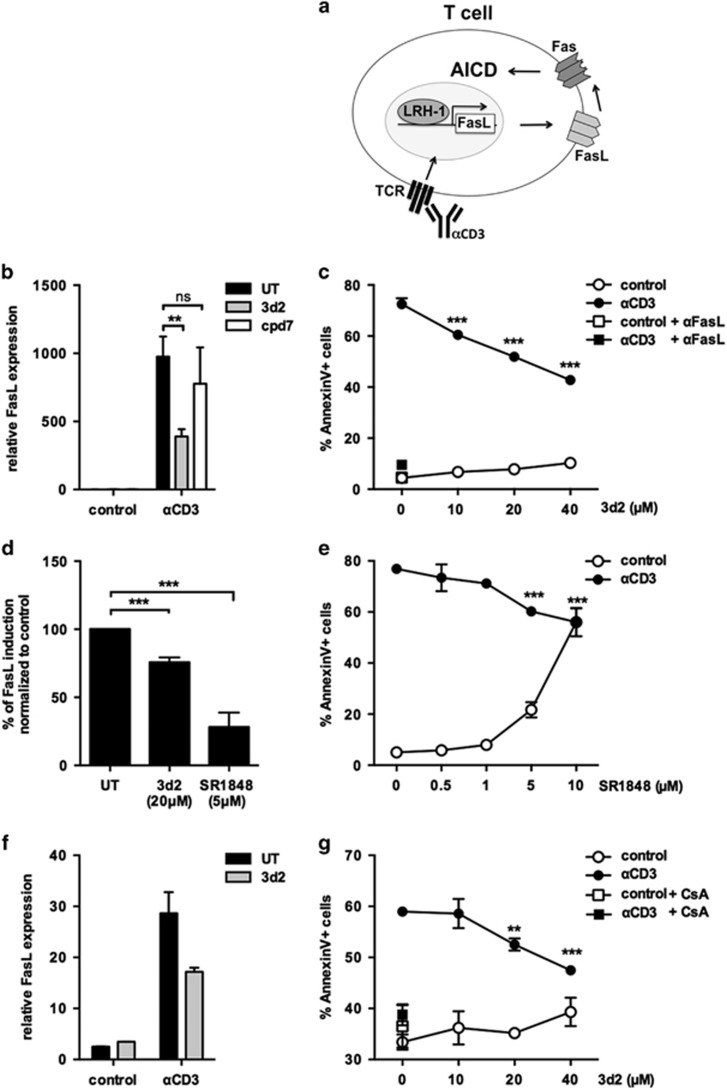
LRH-1 inhibition blocks activation-induced FasL expression and associated T-cell suicide. (**a**) Schematic presentation of FasL-mediated AICD after T-cell receptor activation (TCR) by anti-CD3 antibody (αCD3). (**b**) A1.1 cells were pre-incubated for 2 h with control buffer (UT) or 20 *μ*M 3d2 or cpd7, and then stimulated with plate-bound anti-CD3 (1 *μ*g/ml) for 6 h. FasL mRNA was assessed by quantitative PCR. Mean values of triplicates±S.D. of three independent experiments are shown (unpaired *t*-test; **P<0.01; ns, not significant). (**c**) A1.1 cells were pre-incubated for 2 h with 3d2 at indicated concentrations or 5 *μ*g/ml FasL antibody, followed by stimulation with plate-bound anti-CD3 (1 *μ*g/ml). After 18 h, cells were stained with Annexin V-FITC and analyzed by flow cytometry. Mean values of triplicates±S.D. of a representative experiment (*n*=3) are shown (one-way ANOVA ***P<0.001). (**d**) A1.1 cells pre-incubated for 2 h with control buffer (UT), 20 *μ*M 3d2 or 5 *μ*M SR1848, and stimulated with plate-bound anti-CD3 (1 *μ*g/ml) for 6 h. FasL mRNA expression was assessed by quantitative PCR. Mean values of triplicates±S.D. of a representative experiment (*n*=2) are shown (unpaired *t*-test ***P<0.001). (**e**) A1.1 cells were pre-incubated for 2 h with indicated concentrations of SR1848, followed by stimulation with plate-bound anti-CD3 (1 *μ*g/ml). After 18 h, cells were stained with Annexin V-FITC and analyzed by flow cytometry (one-way ANOVA ***P<0.001). (**f**) Primary murine T-cell blasts were pre-treated with control buffer (UT) or 3d2 (20 *μ*M), and stimulated with plate-bound anti-CD3 (1 *μ*g/ml) for 4 h. FasL mRNA expression was determined by quantitative PCR. Mean values of triplicates±S.D. are shown. (**g**) Primary murine T-cell blasts were pre-incubated for 2 h with indicated concentrations of 3d2 or 100 nM CsA, and then stimulated with plate-bound anti-CD3 (1 *μ*g/ml). After 6 h, cells were stained with Annexin V-FITC and analyzed by flow cytometry. Mean values of triplicates±S.D. of a representative experiment (*n*=2) are shown (one-way ANOVA **P<0.01; ***P<0.001)

Restimulation of previously activated T cells results in cell-autonomous FasL/Fas-mediated suicide,^4^ which is referred to as AICD (Figure 5a). Hence, FasL/Fas-mediated apoptosis contributes to peripheral T-cell homeostasis.^7^ We thus investigated how inhibition of LRH-1 and associated regulation of FasL expression affects AICD in T-cell hybridomas and primary T cells. The results demonstrate that FasL mRNA expression was highly induced in the murine T-cell hybridoma cell line A1.1 when stimulated with immobilized anti-CD3 (Figure 5b). Similar to Jurkat lT cells, 3d2 treatment resulted in a strong decrease of activation-induced FasL expression in A1.1 cells as well. In contrast, no inhibition was observed with the structural homolog cpd7 (Figure 5b). Along with this observation, a dose-dependent inhibition of AICD by 3d2 was monitored (Figure 5c).^4^ FasL dependency was confirmed by inhibition of AICD using a blocking anti-FasL antibody.^25^

To further confirm the specificity of 3d2-mediated inhibition of activation-induced FasL expression we employed a more recently published novel synthetic small molecule repressor of LRH-1, SR1848.^26^ In contrast to 3d2, SR1848 has a different mechanism of action as it promotes translocation of LRH-1 from the nucleus to the cytosol. When A1.1 cells were treated with 5 *μ*M SR1848 a strong inhibition of activation-induced FasL expression was observed, which was more pronounced than that observed after 20 *μ*M 3d2 treatment (Figure 5d). Furthermore, SR1848 inhibited AICD significantly (Figure 5e), despite its cell death-promoting activity at higher concentrations.

Similar results were also obtained for the 3d2-mediated inhibition of AICD in primary murine T cells. Spleen cells were activated with concanavalin A (ConA) for 24 h and expanded using interleukin 2 (IL-2) for 4 days in order to generate T-cell blasts. When these primed T cells were restimulated with plate-bound anti-CD3 strong induction of FasL mRNA expression was observed, which was substantially inhibited by 3d2 (Figure 5f). Accordingly, also AICD in these primary murine T cells was inhibited by 3d2 in a dose-dependent manner (Figure 5g). The specificity of this assay was further confirmed by cyclosporine A (CsA)-mediated inhibition of AICD, supporting a role of NFAT-mediated induction of FasL and associated AICD (Figure 5g).^27^

### LRH-1 inhibitor 3d2 reduces FasL-mediated cytotoxicity

**Figure 6:**
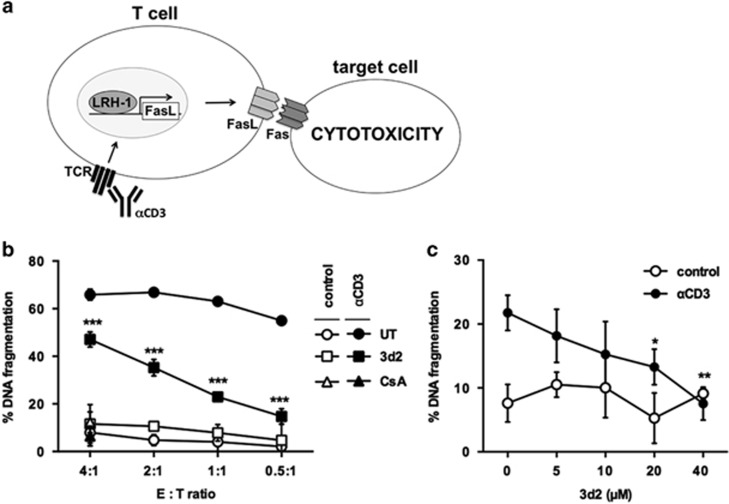
LRH-1 inhibitor 3d2 prevents FasL-mediated cytotoxicity. (**a**) Schematic presentation of the FasL-mediated cytotoxicity assay. (**b**) A1.1 cells were pre-incubated for 2 h with control buffer (UT), 20 *μ*M 3d2 or 100 nM CsA, then stimulated with plate-bound anti-CD3 (3 *μ*g/ml), and co-cultured with Fas-sensitive target cells at indicated effector/target ratios. The percentage of DNA fragmentation in target cells was assessed after 18 h. Mean values of triplicates±S.D. of a representative experiment (*n*=3) are shown (multiple *t*-test ***P<0,001). (**c**) Primary murine T-cell blasts were pre-incubated for 2 h with indicated concentrations of 3d2, and then stimulated with plate-bound anti-CD3 (3 *μ*g/ml). T cells were co-cultured with Fas-sensitive target cells at an effector/target ratio of 1:1. DNA fragmentation was assessed after 18 h. Mean values of triplicates±S.D. of a representative experiment (*n*=2) are shown (one-way ANOVA *P<0.05; **P<0.01)

The second prominent effector function of FasL involves cell-mediated cytotoxicity. The interaction of membrane bound FasL with the Fas receptor on target cells leads to a caspase cascade activation and ultimately apoptosis (Figure 6a).^28^

This cytotoxic effect on Fas receptor-expressing and Fas-sensitive target cells, such as Jurkat lT cells, was investigated by labeling target cells with ^3^H-thymidin and by measuring apoptosis-induced DNA fragmentation.

When A1.1T cell hybridoma cells were co-cultured at different effector:target ratios no apoptosis induction in target cells was observed. In contrast, activation of A1.1 cells with immobilized anti-CD3 antibody resulted in their rapid activation, expression of FasL and killing of Jurkat lT target cells (Figure 6b). Pretreatment with CsA resulted in a complete inhibition of FasL-mediated cytotoxicity, in line with a prominent role of NFAT in the transcriptional control of activation-induced FasL expression.^27, 29^ Similarly, treatment of activated A1.1 cells with the LRH-1 inhibitor 3d2 significantly inhibited FasL-mediated cytotoxicity (Figure 6b). These findings were confirmed in primary murine T cells. Whereas anti-CD3-activated T cells killed Fas-sensitive Jurkat lT target cells, a dose-dependent inhibition of FasL cytotoxicity by 3d2 was observed (Figure 6c). These data confirm that inhibition of LRH-1 activity by 3d2 blocks activation-induced FasL expression and associated FasL-mediated cytotoxicity.

### LRH-1 inhibitor protects from FasL-mediated liver immunopathology

**Figure 7:**
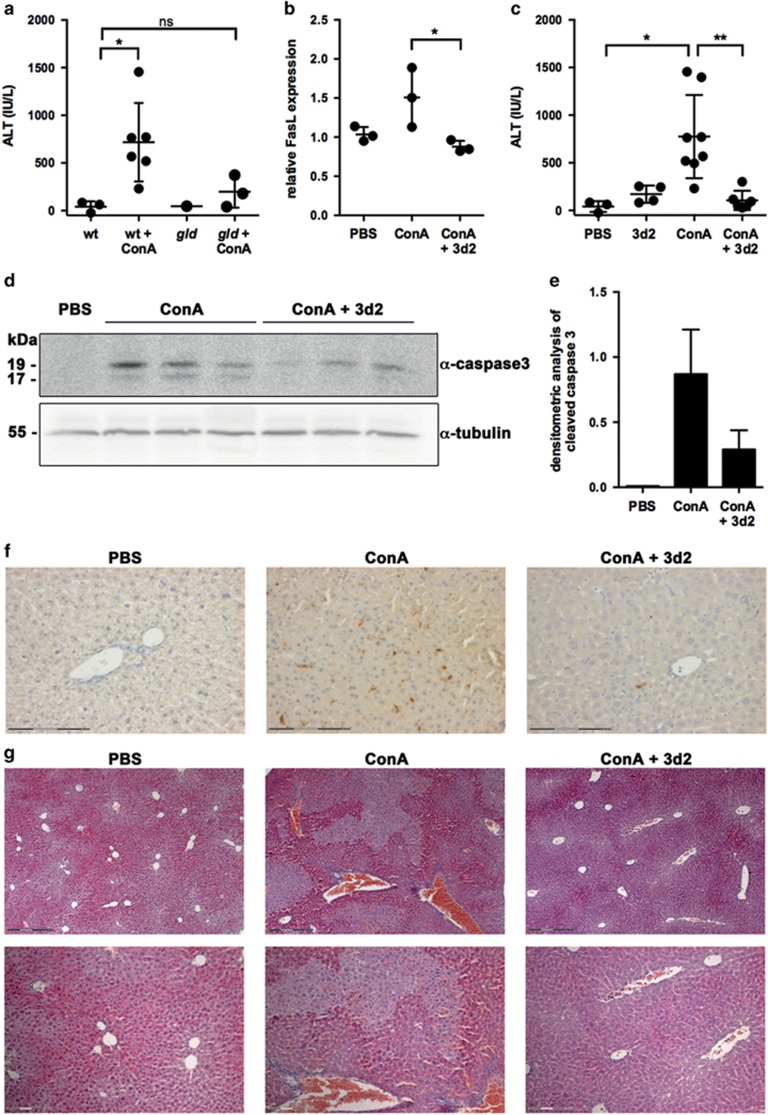
LRH-1 inhibition protects from FasL-mediated liver damage. (**a**) Wild-type (wt) and *gld* mice were injected with ConA, and serum ALT levels were determined after 6 h. Data of individual mice (*n*=1–6) and mean values±S.D. are shown (unpaired *t*-test; *P-value<0.05; ns, not significant). (**b**) Wild-type mice were pre-treated with 3d2 (50 mg/kg body weight) and challenged with ConA or PBS. FasL mRNA expression in liver tissue was measured by quantitative PCR (*n*=3, unpaired *t*-test; *P<0.05). (**c**) Wild-type mice were pre-treated with 3d2 (50 mg/kg body weight), challenged with ConA and serum ALT levels were determined after 6 h (*n*=3–8, unpaired *t*-test; *P<0.05; **P<0.01). (**d**) Mice were treated as in **c** and caspase 3 activation (19 and 17 kDa fragments) in liver tissue was analyzed by western blotting. (**e**) Densitometry analysis of the experiment shown in (**d**). Mean values of triplicates±S.D. are shown. (**f**) Detection of apoptotic, cleaved caspase 3^+^ cells in liver sections from mice treated with PBS, ConA or ConA plus 3d2 (scale bar, 150 *μ*m). (**g**) Hematoxylin/Eosin stainings of liver sections from mice treated with PBS, ConA or ConA plus 3d2 (scale bar, 300 *μ*m, magnification 150 *μ*m)

FasL-mediated cytotoxicity and associated tissue destruction have a critical role in immunopathologies, such as T-cell-mediated hepatitis. Hepatocytes are exquisitely sensitive to Fas-mediated apoptosis and readily die upon treatment with FasL.^30^ We thus aimed to investigate the *in vivo* activity of 3d2 in a mouse model of FasL-mediated liver toxicity. Injection of the lectin ConA into mice induces the activation of liver-homing T cells and NK cells, expression of FasL and consequently killing of sinusoidal endothelial cells and hepatocytes.^31^ This ultimately leads to acute hepatitis, as evidenced by areas of extensive tissue damage in the liver, and increased serum transaminases (Figures 7a, c, f, g).^32^ FasL dependency of ConA-induced hepatotoxicity was confirmed by the reduced liver damage of FasL mutant *gld* mice (Figure 7a).^5^

Although the use of 3d2 has been reported previously in zebrafish,^33^ it had never been applied so far *in vivo* in mammals to inhibit LRH-1-regulated processes. Even though the liver expresses high levels of LRH-1, mice injected with 50 mg/kg body weight 3d2 alone tolerated 3d2 well, and only a mild increase in serum transaminases was observed (Figure 7c). Importantly, while ConA treatment alone resulted in extensive liver damage as evidenced by a profound increase in serum transaminases, caspase 3 activation, apoptosis and histological liver damage, pretreatment of mice for 1 h with 3d2 almost completely abrogated all signs of liver damage and hepatitis (Figures 7c–g). Furthermore, in line with the *in vitro* experiments described above, this protective effect of 3d2 appears to be mediated by inhibition of LRH-1-regulated FasL expression and associated liver damage, as ConA-induced FasL expression in liver tissue was completely reversed by 3d2 (Figure 7b). In order to exclude an inhibitory effect of 3d2 on FasL-mediated apoptosis in hepatocytes, we also analyzed the effect of 3d2 on FasL-induced cell death in isolated and *ex vivo* cultured hepatocytes. Interestingly, while 3d2 alone promoted only minimal cell death it did not inhibit FasL-induced hepatocyte apoptosis (Supplementary Figure 1).

The presented data indicate that LRH-1 regulates activation-induced FasL expression in T cells, and that pharmacological inhibition of LRH-1 has a potential in therapeutic applications on FasL-mediated liver immunopathology.

## Discussion

It is currently widely accepted that LRH-1 is a transcriptional master regulator involved in a large variety of different processes.^16^ Importantly, LRH-1 plays a crucial role in the regulation of inflammation and immune responses in the intestinal mucosa. It regulates not only the intestinal stem cell proliferation and contributes thereby to the intestinal epithelial barrier integrity, but also induces the expression of steroidogenic enzymes and associated synthesis of immunoregulatory glucocorticoids.^17^ While analyzing the role of LRH-1 in pancreatic cancer Benod *et al.* coincidentally detected LRH-1 expression in infiltrating immune cells,^34^ but first direct evidence of LRH-1 expression in hematopoietic cells was provided by Lefèvre and colleagues describing a role for LRH-1 in IL-13-induced macrophage polarization.^35^

In this study, we further extended the investigation on the role of LRH-1 in immune cells, and show for the first time the expression of LRH-1 in primary and secondary lymphoid tissues, and in mature T cells (Figures 1a and b). Furthermore, we provide first evidence that LRH-1 regulates the expression of a critical T-cell effector molecule, that is, FasL. FasL-induced apoptosis is involved in a variety of different processes, but most notably the regulation of T-cell homeostasis and cytotoxicity. Accordingly, FasL mutant *gld* mice show defects in T-cell homeostasis, severe lymphoproliferative disorders and autoimmune diseases.^5^ Furthermore, FasL is recognized as an important cytotoxic effector molecule, particularly involved in the induction of tissue damage during immunopathological disorders, such as acute Graft-versus-Host disease^11^ and hepatitis, as shown here (Figure 7a).^32^

We here provide evidence that LRH-1 regulates *FASLG* transcription via direct interaction with the *FASLG* promoter. Overexpression of LRH-1 increased *FASLG* promoter activity, whereas deletion of *in silico* predicted binding sites resulted in a significant reduction of LRH-1-driven or activation-induced *FASLG* promoter activity (Figure 2c). Furthermore, specific binding of LRH-1 to the promoter was confirmed by ChIP analysis (Figure 2b). Although we identified two putative binding sites only mutation of BS1 resulted in a significant inhibition of LRH-1-induced *FASLG* promoter activity (Figure 2d), suggesting that BS2 is either not important or compensated by BS1. This is in as much interesting as we were able to detect LRH-1 binding to the *FASLG* promoter using BS2-specific primers, whereas detection of BS1 was inconsistent. These at a first glance controversial results may however be explained by the fact that these putative binding sites are only 300 base pairs apart, but the fragmentation of the chromosomal DNA results in fragments of 250 to 1000 base pairs. Thus, specific co-immunoprecipitation of BS1 may also result in DNA fragments, which contain both binding sites. As PCR detection of BS2 appears to be more sensitive than that of BS1, BS2 instead of BS1 was preferentially detected by PCR (Figure 2d). Nonetheless, these data indicate that LRH-1 specifically binds to the *FASLG* promoter and that mutation of LRH-1 binding sites results in reduced *FASLG* promoter activity.

The mutation of both putative LRH-1 binding sites did not completely abrogate LRH-1 overexpression-induced *FASLG* promoter activity. This either suggests that there additional so far unrecognized LRH-1 binding sites within the *FASLG* promoter, or that LRH-1 might also have a more indirect role in the regulation of FasL expression. LRH-1 is known to regulate cell proliferation via the transcriptional regulation of cyclin D1 and E1,^36^ and c-Myc.^34^ In previous studies we demonstrated an interesting correlation between proliferation and FasL expression.^13, 14, 37^ Most notable is the fact that activation of resting T cells results in only low levels of FasL, whereas restimulation of primed proliferating T cells causes rapid and massive induction of *FASLG* transcription. This phenomenon likely ensures that FasL is not expressed in the lymph nodes and the spleen upon activation of resting or naive T cells, but only when they become reactivated at the effector sites. This observation is related to the role of c-Myc and cyclin B1/Cdk1 in the regulation of *FASLG* transcription,^13, 37^ and could likely extend to LRH-1. *FASLG* transcription is regulated by activation- and stress-signal induced factors like NFAT, NF*κ*B, AP-1 and EGR-2 and -3, while transcription factors like c-Myc and likely also LRH-1 may rather have a modulating and enhancing effect, and link *FASLG* transcription to proliferation. Thus, while LRH-1 on one hand directly regulates *FASLG* transcription via specific binding sites in the promoter, it may also indirectly control FasL expression via the regulation of c-Myc.^34, 36^

Unfortunately, testing the role of LRH-1 in the transcriptional control of FasL in a genetic mouse model is hampered by the fact that embryonic deletion of LRH-1 results in lethality.^20^ We thus aimed to exploit the possibilities of pharmacological inhibition to study the role of LRH-1 in FasL expression in T cells. Of interest, pharmacological inhibition of LRH-1 by the inhibitors 3d2 and more pronounced by SR1848 resulted at high doses in extensive cell death induction (Figures 5c, e, g), suggesting a role of LRH-1 in the regulation of T-cell survival by a yet unknown mechanism. Nonetheless, low doses of both inhibitors as well as the inactive control substance cpd7 proved to be useful to investigate the effects of pharmacological intervention on LRH-1-regulated *FASLG* transcription and associated T-cell effector functions. Thus, we report here for the first time the use of the LRH-1 inhibitor 3d2 in a mammalian *in vivo* model of FasL-induced liver damage. ConA injection resulted in the rapid induction of FasL expression and associated liver damage, which was almost completely blocked in *gld* mice, confirming a major role of FasL in this immunopathology. Importantly, 1 h pretreatment of mice with 3d2 significantly resulted in a strong protection from ConA-induced liver damage, as seen in reduced serum transaminase levels and reduced liver cell death (Figures 7c–g). The observation that 3d2 did not inhibit FasL-induced hepatocyte cell death *in vitro* (Supplementary Figure 1) strongly suggests that the inhibitory effect of 3d2 on ConA-induced hepatitis is mediated by inhibition of FasL expression in T cells rather than inhibiting FasL-induced apoptosis in hepatocytes.

These findings demonstrate that LRH-1 is indeed an accessible target for pharmacological intervention in T cells. Most notably, LRH-1 inhibition in T cells appears to be without any obvious side effects on the liver, which expresses high levels of LRH-1. Mice injected with 50 and 100 mg/kg body weight 3d2 alone only showed a very mild increase of serum transaminases (Figure 7c, and data not shown) and no increase in serum TNF levels (data not shown). The relatively low LRH-1 expression in T cells compared with its abundant expression in hepatocytes may provide a therapeutic window. Low doses of 3d2 could allow efficient inhibition of LRH-1 and associated effector functions in T cells, while having only a limited inhibitory effect in hepatocytes and thereby not disturbing vital functions of LRH-1 in the liver. Thus, pharmacological inhibition of LRH-1 may represent an interesting therapeutic option in the treatment of T-cell-dependent and FasL-mediated immunopathologies, such as hepatitis and acute Graft-versus-Host disease.

## Materials and Methods

### Reagents

Phorbol 12-myristate 13-acetate (PMA) and ionomycin were purchased from Enzo Life Sciences (Lörrach, Germany). The LRH-1 inhibitor 3d2 and the control substance compound 7 (cpd7) were re-synthesized according the publication of Benod *et al.*^23^ at ChemBridge Corp (San Diego, CA, USA). The LRH-1 inhibitor SR1848, Cyclosporin A (CsA) and Concanavalin A (ConA, Jack bean, Type IV) were obtained from Sigma-Aldrich (Steinheim, Germany). Hamster anti-mouse FasL antibody (MFL3), anti-mCD4-FITC and anti-mCD8-PE were obtained from BD Biosciences (Heidelberg, Germany). Hamster anti-mouse CD3ɛ antibody (clone 145-2C11) was purified from cell culture supernatant and coated to tissue culture plates in 50 mM Tris–HCl pH 9.0 at 4 °C overnight.

### Mice

Wild-type C57BL/6 mice and *gld* (generalized lymphoproliferative disease) mice with a natural mutation in the *FasL* gene, aged 8–12 weeks were used for *ex vivo* and *in vivo* experiments. Mice were bred in the animal facility of the University of Konstanz, and were accustomed to a 12 h light/dark cycle with free access to food and water. All animal experiments complied with animal experimentation regulations of Germany and were approved by the Ethics Review Committee of the regional council.

### Cell culture

The human embryonic kidney fibroblast cell line HEK 293T cells was obtained from American Type Culture Collection (ATCC) and was maintained in Dulbecco's Modified Eagle's Medium. The murine T-cell hybridoma A1.1 cells^31^ and the human leukemic T lymphocyte Jurkat lT cells^38^ were cultured in RPMI-1640 Medium. All cell culture media were supplemented with 10% FCS and 30 *μ*g/ml gentamycin.

Murine T-cell blasts were generated by stimulating isolated splenocytes with 1 *μ*g/ml ConA in RPMI-1640 medium supplemented with 10% FCS, 2 mM l-glutamine, 50 *μ*M 2-mercaptoethanol and 30 *μ*g/ml gentamycin for 1 day. Thereafter, cells were washed to remove the lectin, and were cultured with 100 units/ml recombinant IL-2 (Proleukin, Prometheus, Vevey, Switzerland) for additional 4 days to generate T-cell blasts. All other reagents were obtained from Sigma-Aldrich (Steinheim, Germany).

### Cell sorting

For cell sorting splenocytes were stained with anti-CD4-FITC and anti-CD8-PE antibodies diluted in PBS. Afterwards, cells were washed once and sorted on a FACSAria III using FACSDiva software (BD Heidelberg, Germany).

### Plasmids

The human FasL luciferase reporter construct (HFLP-Luc) containing 1.2 kb of the *FASLG* promoter, and the empty control plasmid HsLuc have been described previously.^38^ HFLP-Luc with single or double mutated LRH-1 binding sites in the *FASLG* promoter (HFLP mut BS1, HFLP mut BS2, HFLP mut BS1+2) were generated by site-directed mutagenesis using a kit from Stratagene (Quick-Change, Agilent) with primers shown in Table 1. The putative LRH-1 binding sites and in the *FASLG* promoter and their mutation are depicted in Figure 2a.

The LRH-1 reporter containing 5 copies of the LRH-1 binding motive of the human *SHP* promoter in the pGL3 basic plasmid (Promega, Mannheim, Germany) has been described previously.^39^ The Myc/6xHis-tagged LRH-1 expression plasmid was generated by cloning human LRH-1 into a pcDNA3.1 Myc/His expression vector (Invitrogen). The FLAG-tagged LRH-1 expression plasmid was generated by exchanging the Myc/His tag with a 3xFLAG epitope tag. A β-galactosidase expression plasmid was used to normalize transfection efficiency (Invitrogen).

### Transfection

One day before transfection, 3 × 10^5^ HEK 293T cells were seeded into a 6-well plate. Cells were transfected with a total of 1 μg of plasmid DNA using the calcium phosphate precipitation method for 6 h. Jurkat lT cells were seeded one day prior transfection at a density of 3 × 10^5^ cells/ml. A total of 1 × 10^6^ cells were transfected with 2 μg of plasmid DNA in a Gene Pulser Cuvette (BioRad, München, Germany) by electroporation using an Amaxa Nucleofector Device (Lonza, Basel, Switzerland). Electroporated cells were pooled in complete medium and seeded into a tissue culture dish for further experiments.

### Reporter assays

HEK 293T cells and Jurkat lT cells were transiently transfected with expression and luciferase reporter plasmids. Co-transfection of β-galactosidase expression plasmid served as a normalization. One day after transfection, cells were either control treated or treated with different concentrations of the LRH-1 inhibitors and controls for 2 h, prior to the stimulation with PMA (50 ng/ml) and ionomycin (500 ng/ml) for 16 h. Cells were then lysed, and luciferase and β-galactosidase activity was measured in cell lysates.^23^

### Chromatin immunoprecipitation

Jurkat lT cells (1 × 10^7^ cells) were transfected with the FLAG-tagged LRH-1 expression vector or pcDNA3.1 as empty vector control. After 48 h cells were cross-linked with 1% formaldehyde for 10 min at 37 °C. The cells were then lysed with buffer A (10 mM HEPES, 10 mM KCl, 0.1 mM EDTA, 0.1 mM EGTA, 1 mM DTT, 0.5 mM phenylmethylsulfonide fluoride) on ice for 15 min to obtain the cytosolic fractions. Nucleic pellets were lysed with nuclei lysis buffer (50 mM Tris–HCl pH 8.0, 10 mM EDTA, 1% SDS, phenylmethylsulfonide fluoride, complete Protease Inhibitor Cocktail Tablets (Roche)) on ice for 10 min. Samples were sonicated for 26 cycles (for 30 s each, high intensity) using a Bioruptor Plus (Diagenode, Seraing, Belgium). The supernatant was diluted in immunoprecipitation dilution buffer (16.7 mM Tris–HCl, 167 mM NaCl, 1.2 mM EDTA, 0.01% SDS, 1.1% Triton X-100) and incubated overnight at 4 °C with 5 *μ*g mouse anti-FLAG antibody or control mouse IgG antibody (Sigma-Aldrich, Steinheim, Germany). Antibodies were then immunoprecipitated using Magna CHIP Protein G magnetic beads (Merck Millipore, Darmstadt, Germany) while rotating for 2 h at 4 °C. Beads were washed in high-salt buffer (50 mM HEPES pH 7.9, 500 mM NaCl, 1 mM EDTA, 0,1% SDS, 1% Triton X-100, 0,1% deoxycholate). DNA was purified using the IPure kit (Diagenode) and magnetic beads according to the manufacturer's protocol. The following amplification of the putative LRH-1 binding sites in the *FASLG* promoter or the *SHP* promoter (positive control) was performed by PCR with primers stated in Table 1. PCR products were then separated and analyzed by agarose gel electrophoresis.

### Reverse transcription and real-time PCR

For RNA isolation cells were lysed in 1 ml peqGOLD TriFast reagent (PeqLab, Erlangen, Germany). Tissue samples were homogenized in 1 ml TriFast using the TissueLyser II (Quiagen, Hilden, Germany). RNA was isolated according to the manufacturers protocol. One microgram of RNA was reverse transcribed using a High-Capacity cDNA Reverse Transcription Kit (Applied Biosystems, Foster City, CA, USA) and cDNAs were used for quantification of gene expression by quantitative PCR using FAST SYBR Green Master Kit and a StepOnePlus Real-time PCR system (Applied Biosystems) with primers stated in Table 1.

### Activation-induced T-cell death

Four days after ConA stimulation and IL-2-induced expansion, murine T-cell blasts were purified using a Histopaque-1077 (Sigma-Aldrich) gradient to remove dead cells. Viable primary T cells or A1.1 cells were pre-treated with 3d2, SR1848, CsA or anti-FasL antibody for 2 h and cultured for 6 h at 3 × 10^5^ cells/well in anti-CD3 antibody-coated 96-well plates. Subsequently, cells were harvested, stained with Annexin V-FITC in binding buffer (10 mM HEPES pH 7.4, 150 mM NaCl, 5 mM KCl, 1 mM MgCl_2_, 1.5 mM CaCl_2_), and were analyzed using LSR Fortessa (BD Biosciences). A population of 10 000 cells was analyzed for each sample. The number of Annexin V^+^ apoptotic cells as a percentage of total cells was determined using FlowJo software (version 10).

### Cytotoxicity assay

Jurkat lT target cells were labeled with 10 μCi/ml [methyl-^3^H]-Thymidine (Hartmann Analytic, Braunschweig, Germany) for 3 h in a humidified incubator with 5% CO_2_ at 37 °C. Primary T-cell blasts or A1.1 cells (effector cells) were serially diluted in a 96-well plate coated with anti-CD3 antibody (1 μg/ml) and treated with 3d2 or CsA.

Labeled target cells were washed twice, resuspended in complete medium, and 100 μl cell suspension (20 000 cells) were added to the plate and incubated with the effector cells for 18 h. Afterwards, ^3^H-Thymidine-labeled target cell DNA was harvested onto glass fiber filters using Omnifilter-96 cell harvester (Perkin, Rodgau, Germany Elmer), and radioactivity was measured using a TopCount Microplate Scintillation Counter (PerkinElmer, Rodgau, Germany). Subsequently, DNA fragmentation was calculated % DNA fragmentation=100 × (1−cpm experimental sample sample/cpm targets only).

### ConA-induced hepatitis

ConA was diluted in sterile endotoxin-free PBS. Male mice were pre-treated with 3d2 (50 mg/kg body weight, dissolved in 10% DMSO in PBS) by i.p. injection 1 h before the ConA injection (i.v.10 mg/kg body weight). Six hours after the challenge, mice were sacrificed and serum samples were collected. Liver samples were snap-frozen in liquid nitrogen and then stored at −80 °C for RNA isolation and protein assays, or fixed in formalin for histology and cleaved caspase 3 immunohistochemistry. Alanine aminotransferase (ALT) in the serum samples was measured using a colorimetric kit (Teco Diagnostics, Anaheim, CA, USA) according to the manufacturer's instructions.

### Western blot analysis

Frozen liver tissue was homogenized in NP-40 lysis buffer (150 mM NaCl, 50 mM Tris, pH 7.6, 1 mM EDTA and 1% NP-40) using a tissue lyser (Quiagen) and protein concentrations were determined. Samples were then separated on a denaturing 12% SDS-PAGE gel. After transfer to polyvinylidene difluoride membranes (PVDF) (Roche), caspase activation was detected using a rabbit polyclonal anti-caspase 3 antibody (Cell Signaling, Davers, MA, USA) or mouse anti-tubulin (Sigma-Aldrich, Steinheim, Germany) as loading control.

### Histology and immunohistochemistry

Briefly, tissue sections were generated from formalin-fixed and paraffin-embedded liver tissue. For histological analysis they were counter-stained with hematoxilin and eosin. Cleaved caspase 3 as a marker of apoptotic cells was detected by immunohistochemistry as described previously.^40^

### Statistical analysis

Student's *t*-test, multiple *t*-test and ordinary one-way ANOVA were performed on Prism7 software (GraphPad Software, La Jolla, CA, USA) to define significant differences between experimental groups. A *P*-value of <0.05 was regarded significant.

## Figures and Tables

**Table 1:** Primer sequences

*qPCR primer:*		
Human GAPDH	for	5′-ATG GAG AAG GCT GGG GCT CA-3′
	rev	5′-TCT CCA TGG TGG TGA AGA CA-3′
Human LRH-1	for	5′-GGG CAA CAA GTG GAC TAT TC-3′
	rev	5′-CCA GCT GGA AGT TTT CAA GG-3′
Human FasL	for	5′-GGC CTG TGT CTC CTT GTG AT-3′
	rev	5′-TGC CAG CTC CTT CTG TAG GT-3′
Murine GAPDH	for	5′-CGT CCC GTA GAC AAA ATG GT-3′
	rev	5′-TCT CCA TGG TGG TGA AGA CA-3′
Murine β-actin	for	5′-TAT TGG CAA CGA GCG GTT CC-3′
	rev	5′-GCA CTG TGT TGG CAT AGA GG-3′
Murine LRH-1	for	5′-TTG AGT GGG CCA GGA GTA GT-3′
	rev	5′-ACG CGA CTT CTG TGT GTG AG-3′
Murine FasL	for	5′-TTC CAC CTG CAG AAG GAA C-3′
	rev	5′-TAA ATG GGC CAC ACT CCT C-3′
		
*ChIP-PCR primer:*		
ChIP HFLP BS1	for	5′-CTG TGG GTT CAG TGG TTT G-3′
	rev	5′-AGT CAT GGC CAG AGA AGT C-3′
ChIP HFLP BS2	for	5′-GGA GCA GTT CAC ACT AAC AG-3′
	rev	5′-AAA CAC CCA CTC GCT TTG-3′
ChIP SHP	for	5′-AAC ACT TCT GCC CAG ATC AC-3′
	rev	5′-GCC TCT TCC TAA GGC TAG ATT C-3′
		
*Site-directed mutagenesis primer:*		
mut BS1	for	5′-ATT ATG GTG ATC GGC TAA TCA GGG TAA ATG GTA GTT G-3′
	rev	5′-CAA CTA CCA TTT ACC CTG ATT AGC CGA TCA CCA TAA T-3′
mut BS2	for	5′-GCT ATA CCC CCA TGC TGA TTA GCT CTG CAG GAT CCC-3′
	rev	5′-GGG ATC CTG CAG AGC TAA TCA GCA TGG GGG GTA TAG C-3′

Abbreviations: for, forward; rev, reverse.
