# Supplementary material for: Liver receptor homolog-1 (NR5a2) regulates CD95/Fas ligand transcription and associated T-cell effector functions
Source: Cell Death Dis. 2017 Apr 13;8(4):e2745–. doi: 10.1038/cddis.2017.173 (PMC5477591; doi:10.1038/cddis.2017.173)
Supplement: Supplementary Information [file cddis2017173x1.docx]

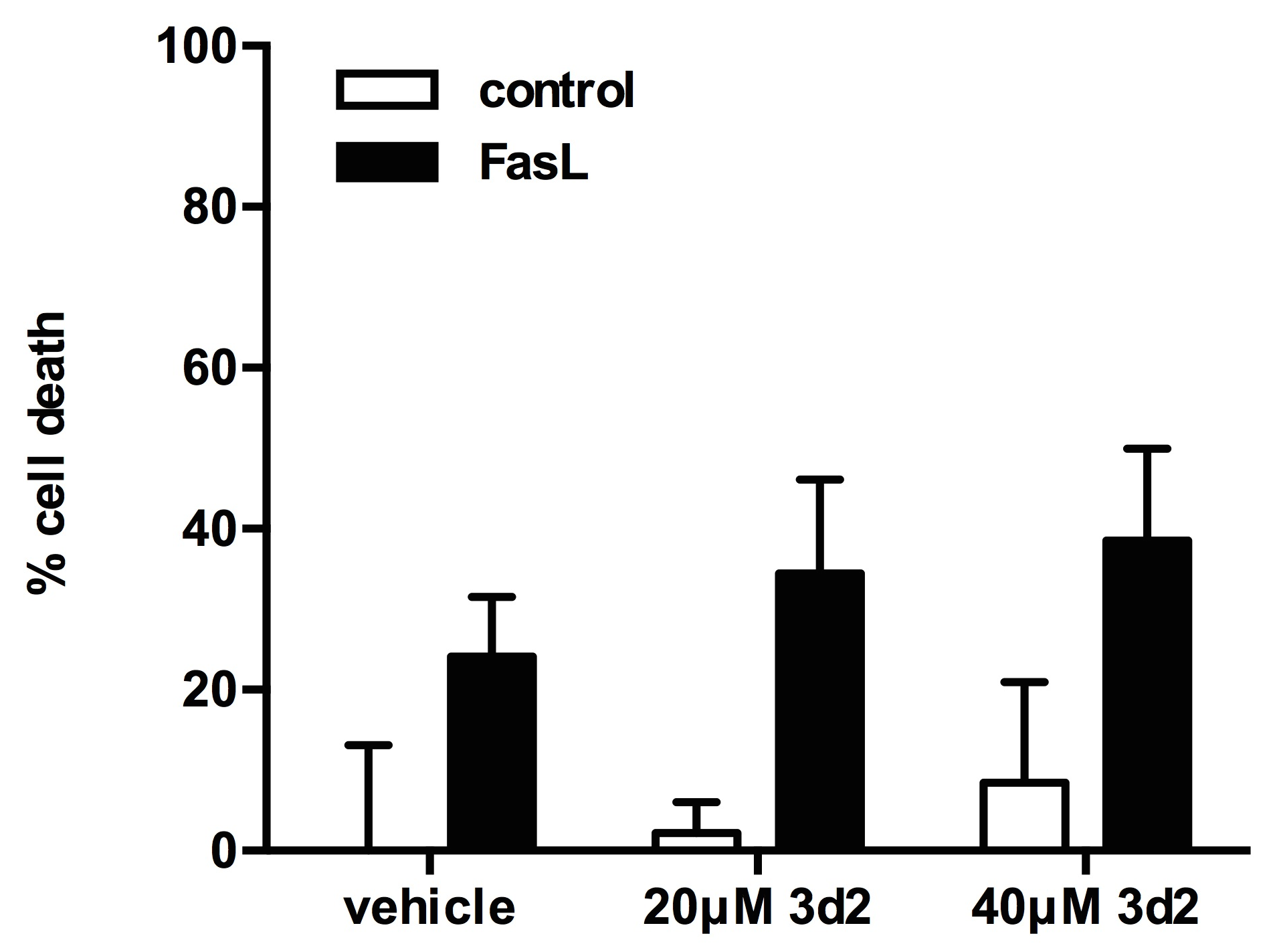


**Supplementary Figure 1: *3d2 does not inhibit FasL-induced hepatocyte cell death.***

Primary hepatocytes were isolated from C57Bl/6 wild type mice according to previously published methods (1) and cultured in 96-well plates. Cells were then pretreated with indicated doses of 3d2 for 1 h prior to induction of cell death by 40 ng/ml recombinant FasL-Fc fusion protein for additional 4 h (1). Cell death was analyzed by MTT assay (1).

1. Corazza, N., S. Jakob, C. Schaer, S. Frese, A. Keogh, D. Stroka, D. Kassahn, R. Torgler, C. Mueller, P. Schneider, and **T. Brunner.** 2006. TRAIL receptor-mediated JNK activation and Bim phosphorylation critically regulate Fas-mediated liver damage and lethality. ***J Clin Invest.*** 116:2493-2499.
